# P-449. Healthcare Burden of Venous Thromboembolism (VTE) among People Living with Human Immunodeficiency Virus (PLHIV): A Nationwide Inpatient Data Analysis (2019-2021)

**DOI:** 10.1093/ofid/ofae631.649

**Published:** 2025-01-29

**Authors:** Chef Stan L Macaraeg, Kim Abbegail Aldecoa, Cunegundo Vergara

**Affiliations:** University of Connecticut School of Medicine, Hartford, Connecticut; Trinity Health Oakland/Wayne State University, Pontiac, Michigan; Hartford Healthcare, Hartford, Connecticut

## Abstract

**Background:**

Venous Thromboembolism (VTE) imposes a substantial economic burden, estimated to incur almost $10 billion each year. People Living with Human Immunodeficiency Virus (PLHIV) face an almost two- to tenfold higher risk of developing VTE compared to the general population. However, limited data exists on the healthcare burden of VTE among PLHIV. The study investigated the healthcare burden of VTE among PLHIV compared to non-HIV patients. Additionally, the study examined the difference in sociodemographic factors between the two groups.

**Methods:**

We analyzed data from the National Inpatient Sample database from 2019 to 2021 to identify all hospitalizations with a primary diagnosis of Acute Venous Thromboembolism. Subsequently, we categorized patients into HIV and non-HIV groups. Stata 18.0 was utilized for analysis, with a p-value < 0.05 indicating statistical significance. Hospital costs were adjusted for inflation using the US Consumer Price Index.

**Results:**

Among the 821,494 hospitalizations for VTE, 2230 patients had HIV infection. HIV patients were significantly younger (52.5 vs. 63.4 years) and predominantly male, Black (56.3% vs. 20.0%), and Hispanic (11.3% vs. 7.2%) individuals. The HIV group exhibited longer hospital stays (6.23 vs. 4.37 days) and incurred higher total hospital costs ($19,117 vs. $14,115) after adjusting for age and sex. Notably, there was a positive correlation between length of stay and hospital costs (r=0.68, p< 0.001). The incidence of AKI and pneumonia significantly prolonged hospital stays in the HIV subgroup (aOR: 2.96, 95% CI: 1.21-4.70; aOR: 2.92, 95% CI: 0.42-5.42). From 2019 to 2021, hospital costs showed a significant increase ($18338, $20330, $24507, p=0.034), while the hospital length of stay displayed an increasing trend but was not statistically significant (mean in days: 5.9, 6.4, 7.0, p=0.147). There was no specific trend observed in the VTE rate each year (0.28%, 0.26%, 0.28%, p=0.7875) among the HIV subgroup.

**Conclusion:**

The healthcare burden of VTE hospitalization is higher among PLHIV. There has been a concerning increasing trend in hospital costs among PLHIV from 2019 to 2021. Future studies should investigate additional factors contributing to this healthcare burden to enhance the cost-effectiveness of care.

**Disclosures:**

**All Authors**: No reported disclosures

